# Plasmonic Field-Effect Transistors (TeraFETs) for 6G Communications

**DOI:** 10.3390/s21237907

**Published:** 2021-11-27

**Authors:** Michael Shur, Gregory Aizin, Taiichi Otsuji, Victor Ryzhii

**Affiliations:** 1 Rensselaer Polytechnic Institute, Troy, NY 12180, USA; 2 Electronics of the Future, Inc., Vienna, VA 22181, USA; 3 Kingsborough College, The City University of New York, Brooklyn, NY 11235, USA; gregory.aizin@kbcc.cuny.edu; 4 Research Institute of Electrical Communication, Tohoku University, Sendai 980-8577, Japan; otsuji@riec.tohoku.ac.jp (T.O.); v-ryzhii@riec.tohoku.ac.jp (V.R.); 5 Institute of Ultra High Frequency Semiconductor Electronics of RAS, 117105 Moscow, Russia

**Keywords:** 6G communications, plasmonic crystals, field-effect transistor arrays, plasma wave instabilities, terahertz detection, terahertz generation, line-of-sight detection, silicon CMOS, travelling wave amplifier, terahertz radiation

## Abstract

Ever increasing demands of data traffic makes the transition to 6G communications in the 300 GHz band inevitable. Short-channel field-effect transistors (FETs) have demonstrated excellent potential for detection and generation of terahertz (THz) and sub-THz radiation. Such transistors (often referred to as TeraFETs) include short-channel silicon complementary metal oxide (CMOS). The ballistic and quasi-ballistic electron transport in the TeraFET channels determine the TeraFET response at the sub-THz and THz frequencies. TeraFET arrays could form plasmonic crystals with nanoscale unit cells smaller or comparable to the electron mean free path but with the overall dimensions comparable with the radiation wavelength. Such plasmonic crystals have a potential of supporting the transition to 6G communications. The oscillations of the electron density (plasma waves) in the FET channels determine the phase relations between the unit cells of a FET plasmonic crystal. Excited by the impinging radiation and rectified by the device nonlinearities, the plasma waves could detect both the radiation intensity and the phase enabling the line-of-sight terahertz (THz) detection, spectrometry, amplification, and generation for 6G communication.

## 1. Introduction

**Figure 1:**
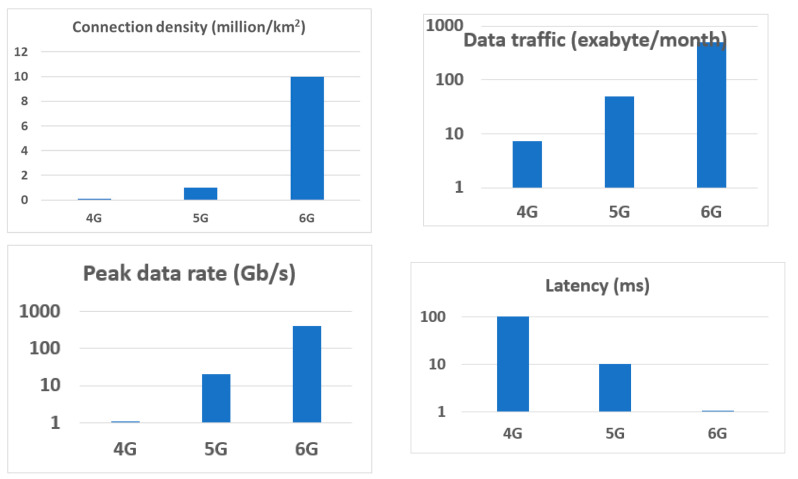
Communication evolution from 4G to 6G.

Within literally one generation, the Internet revolutionized our lives and proved to be a lifesaver during the COVID-19 pandemic. The wireless communication during the pandemic increased about 40%. Teleconferencing increased by about 300% [1]. It was mostly enabled by one material—silicon—and by one device—the field-effect transistor—albeit with a lot of help from germanium, III–V, and some other materials. We now use 4G and emerging 5G technology, but the 6G communications will be another transformational jump in communications (see Figure 1). 6G will raise applications in telemedicine, teleconferencing, defense, industrial controls, cyber security, the Internet of Things, autonomous unmanned cars, robotics and stay-at-home work and conferencing to a much higher level [2,3,4,5,6,7,8,9,10,11]. 6G will expand the wireless high-speed communications to the space, sea, and upper atmosphere.

Analyzing the spectrum of possible pandemic outcomes using the Pandemic Equation [12] shows that the pandemic tail and even possible spikes might still be with us for years to come driven by new emerging COVID-19 virus variants, such as the Delta variant. This makes the planned transition to the 6G communications using the sub-terahertz (sub-THz) range to be even more important. This 300 GHz range has been identified as the range between 252.72 GHz to 321.84 GHz [13].

**Figure 2:**
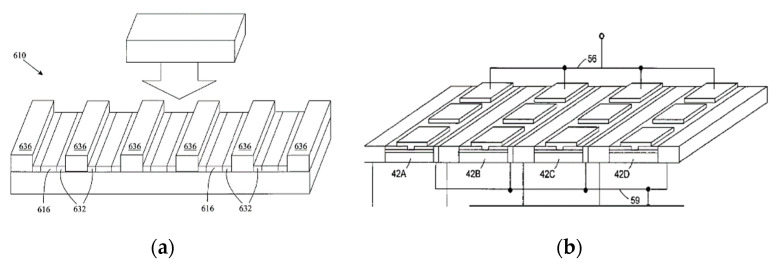
Plasmonic crystal concept: multi finger (grating gate) (from [14]) (**a**) and two-dimensional plasmonic array (from [15]) (**b**).

Among the technologies to support such a transition, the plasmonic crystal technology has the potential to become a winner. A unit cell of such a crystal has small dimensions to support the ballistic or quasi-ballistic transport and plasmonic resonances, while the overall size of the crystal is sufficiently large to efficiently capture or emit a sub-THz or a THz beam (see Figure 2) [14,15,16,17,18,19,20,21,22,23,24,25,26,27,28,29,30,31,32,33].

For example, the critical unit cell dimensions for silicon at room temperature might be on the order of 20 to 50 nm depending on the mobility and electron density. For the 240 to 320 GHz range, the overall dimension of the plasmonic crystal device could be in the millimeter range. Unique circuit applications for the plasmonic crystal devices could range from the line-of-sight detection [34], spectroscopy [35,36], homodyne [37,38,39] or heterodyne detection [40,41,42,43], frequency to digital conversion [44], and travelling wave amplifiers [45].

Plasma waves were first predicted by Tonks and Langmuir in 1929 [46]. In 1952, David Pines and David Bohm introduced the term “plasmon” [47]. The seminal works of Stern and Ferrell [48] and Chaplik [49] considered plasma waves in semiconductors. The promise and demonstrations of the THz generation by unstable resonant plasma waves [50,51,52,53,54,55,56,57,58,59,60], and of the THz detection by both resonant [61,62,63,64,65,66] and decaying [67,68,69,70,71,72,73,74,75,76,77,78,79,80,81,82,83,84] plasma waves has stimulated a lot of interest to this research area and resulted in the demonstration of the THz plasmonic detection in Si [70,71,72,73,74,75], III–V [76,77,78], III–N [79,80,81,82], monolayer, bilayer, and bipolar graphene [64,65,85,86,87,88,89,90]. In this paper, we analyze the applications of this technology for future 6G communications.

## 2. Plasmonic TeraFETs

Field-effect transistors operating in plasmonic regimes and often referred to as TeraFETs have already demonstrated an impressive performance in the sub-THz and THz range (see Table 1).

**Figure 3:**
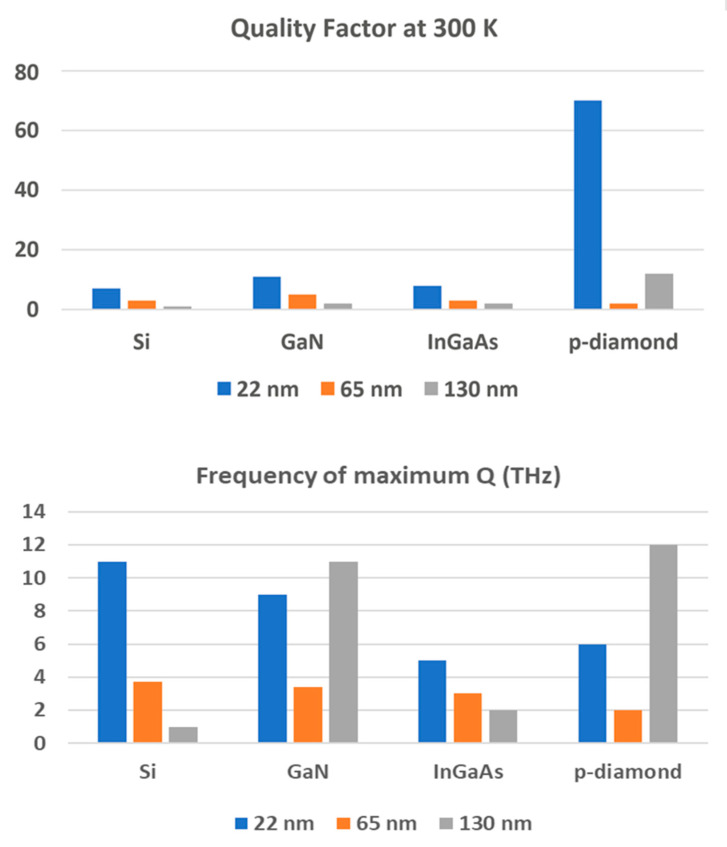
Estimated values of the maximum quality factor Qm, and the frequency at which this value is achieved, fm, for Si, GaN, InGaAs, and p-diamond TeraFETs (data from [92,93]). Parameters used in the calculation: mobilities for Si 0.1450 m^2^/Vs, for GaN 0.2 m^2^/Vs, for InGaAs 1.2 m^2^/Vs, and for p-diamond 0.53 m^2^/Vs; effective masses: for Si 0.19, for GaN 0.23, for InGaAs 0.041, and for p-diamond 0.663.

Grated gate structures [14,15,16,17,18,19,20,21,22,23,24,25,26,27,28,29,30,31,32,33,77,78,89,90,91] demonstrated a better performance compared to single TeraFETs and a promise of THz radiation. Most of the room temperature results are for damped plasma wave detection by field-effect transistors. Figure 3 presents the largest calculated resonant quality factors for the single TeraFETs and the plasmonic frequencies at which these maximum quality factors are obtained.

The quality factor values, *Q*, shown in Figure 3, were obtained using the following equation:

(1) $$Q=\omega_{p}\tau_{eff}$$

Here $\omega_{p}=sk$ is the plasma velocity, k=π/(2L) is the wave vector of the fundamental plasmonic mode for a TeraFET detector, and *s* is the plasma wave velocity:

(2) $$s=v_{th}{\left(\left(1+\exp\left[-qU_{o}/\left(\eta k_{B}T\right)\right]\right)\ln\left[1+\exp\left[qU_{o}/\left(\eta k_{B}T\right)\right]\right]\right)}^{0.5}$$

*U_o_ = U_g_ − U_T_* is the gate voltage swing, *U_g_* is the gate-to-source voltage, *U_g_ − U_T_* is the threshold voltage, *h* is the subthreshold ideality factor, $1/\tau_{eff}=1/\tau_{m}+\nu k^{2}$ is the effective scattering frequency, ν is the electron fluid viscosity, *L* is the channel length, $\tau_{m}=\mu m/q$ is the momentum relaxation time, *m* is the low field mobility, *m* is the electron effective mass, *q* is the electronic charge, *k_B_* is the Boltzmann constant, and *T* is the temperature.

The quality factors in Figure 3 are much smaller than that for the photonic crystals operating in near infrared or visible range and using, for example, gold nanoparticles [94]. Moreover, the values in Table 2 represent the upper bound of the quality factors of the THz TeraFETs that could be achieved. Parasitic elements [95], surface scattering [96], quantum reflection [97] from the contact regions all conspire to reduce the quality factors. Oblique waves were mentioned as another reason for the quality factor reduction [98], which, however, was not confirmed by numerical simulations [99]. For example, the calculation in [91,92] predicts the quality factor for InGaAs at 77 K of 18. In fact, the measured quality factor at 110 K was 1.4 [100]. The resonant behavior for Si TeraFETs at room temperature for the 20 nm NMOS (Q = 7) is predicted for 11 THz, which is a larger frequency that the optical phonon frequency in Si (~8 THz). At such frequency, coupling with lattice vibrations should be accounted for. However, the data in Figure 3 provide the guidance for searching for the resonant plasmonic response at room temperature.

Table 2 compares the performance of THz TeraFET detectors with other THz detectors. As seen, the achieved TeraFET performance is at or above the state-of-the-art. Since the TeraFET technology is still developing, it is expected to become a leading THz detection technology. In addition to fast speed, tunability, and operating in a wide temperature range, possibly the greatest advantage of TeraFETs is the compatibility with the Si CMOS technology. All THz communication components, detectors, generators, and modulators operating in the 300 GHz band could be implemented using Si CMOS TeraFETs. This makes this technology especially appealing for the 6G communication integrated circuits.

The bandwidth is determined by the received power, *P_r_*_,_ the signal-to-noise ratio, *SN*, and the detector noise equivalent power, NEP [104].

(3) $$BW=\frac{P_{r}^{2}}{SN^{2}NEP^{2}}$$

(4) $$P_{r}=\frac{c^{2}G_{r}G_{t}P_{t}}{A{\left(4\pi Rf\right)}^{2}}$$

Here, *P_t_* is the transmitted power, *c* is the speed of light, *G_r_* and *G_t_* is the receiving and transmitting antenna gains, *A* is the propagation loss, *R* is the communication distance, and *f* is the communication frequency. The state-of-the-art Si FET emitters using frequency multiplication generate in the order of a hundred of microwatts power in the 300 GHz range [105,106]. However, the TeraFET output power could be increased using a series connection of TeraFETs [107] and even more by using the plasmonic crystal TeraFETs discussed in the next section with the estimated power output on the order ~100 mW [20]. Calculations using Equations (3) and (4) show that this power might be sufficient if the receiver NEP could be reduced to 0.1 pW/Hz^1/2^ from the current value of 0.5 pW/Hz^1/2^ (see Table 1). As seen from Equations (3) and (4), this increase in the transmitted power enable orders of magnitude increases in the bandwidth and/or in the communication range.

TeraFETs could detect and generate THz radiation. However, their modulation speed is limited by the transistor cutoff frequency, *f_T_* [108]. The upper bound of *f_T_* is given by [108]:

(5) $$f_{To}=\frac{v_{s}}{2\pi L}$$

where *L* is the channel length and *v_s_* is the electron saturation velocity. For silicon longer channel devices, *v_s_* is ~10^5^ m/s and could be ~50% higher in short-channel devices [109]. However, as shown in [110], the maximum modulation frequency peaks at the inverse effective electron relaxation time, which was taken as the electron momentum relaxation time, $\tau_{m}=m\mu /q$, where *m* is the electron effective mass.

The contribution of this effect into *f_T_* could be estimated as follows:

(6) $$f_{T}=\frac{1}{1/f_{To}+\tau_{m}}$$

**Figure 4:**
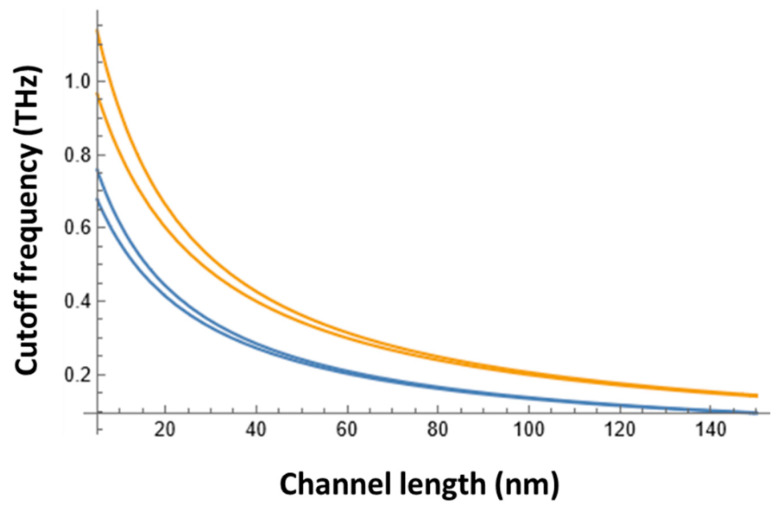
Dependence of the cutoff frequency on the channel length for *v_s_* = 10^5^ m/s (two bottom curves) and *v_s_* = 1.5 × 10^5^ m/s (two top curves) with (bottom line in each set) and without (top line in each set) accounting for the electron momentum relaxation time.

Figure 4 shows dependence of the cutoff frequency on the channel length for *v_s_* = 10^5^ m/s and *v_s_* = 1.5 × 10^5^ m/s with and without accounting for the electron momentum relaxation time. As seen, Si TeraFETs could be modulated at sub-THz frequency, enabling their application for the 300 GHz band 6G communication.

(The bandwidth is $BW\approx 2f_{T}$, since the TeraFETs could be modulated at frequencies on the order of $f_{T}$.)

## 3. Plasmonic Crystals

**Figure 5:**
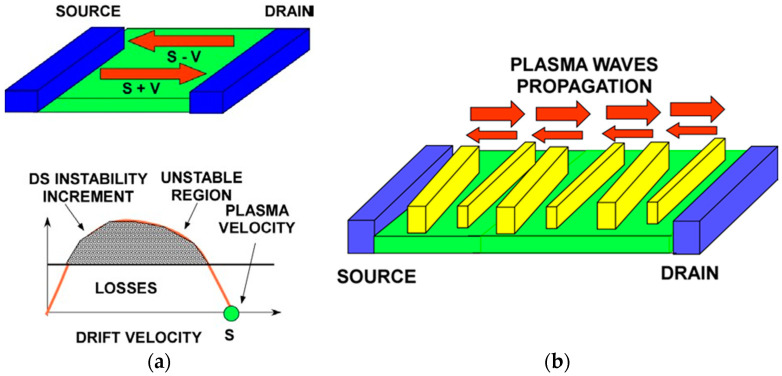
The Dyakonov–Shur (DS) instability increment (**a**) and 1D plasmonic crystal implementation (**b**).

**Figure 6:**
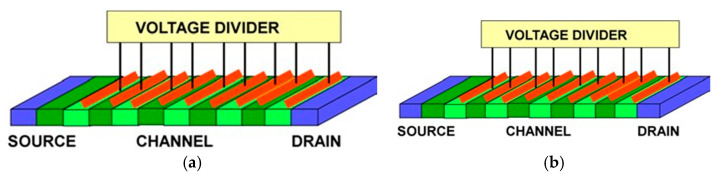
Plasmonic boom: variable electron sheet density (**a**) and variable width structures (**b**).

**Figure 7:**
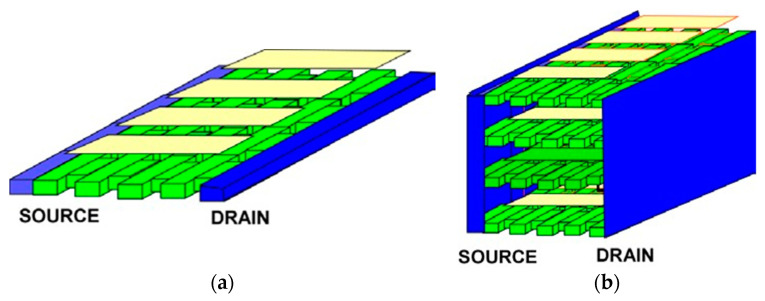
2D (**a**) and 3D (**b**) plasmonic crystals of variable width.

**Figure 8:**
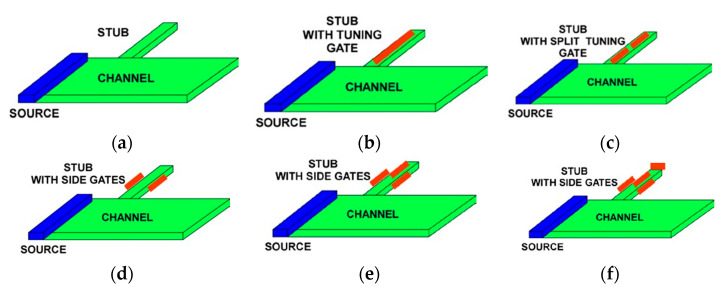
Stubs for tuning: (**a**) ungated stub; (**b**) stub with a single tuning gate; (**c**) stub with a split tuning gate; (**d**) stub with side gates; (**e**) stube with side gates and top gate; (**f**) stub with side gate and two top tuning gates.

Figure 5, Figure 6, Figure 7 and Figure 8 show different plasmonic crystal implementations proposed for detecting, processing, and generating the THz radiation. Figure 5 illustrated the Dyakonov–Shur (DS) instability mechanism [50] that relies on the difference in the reflection coefficients from the channel boundaries due to the differences in the velocities of the plasma waves propagating from the source to the drain (*s* + *v*) and reflected from the drain (*s* − *v*). Here, *v* is the electron drift velocity. The largest increment corresponds to the boundary conditions of the short-circuited source and open drain. Having finite impedances at the source and drain reduces the increment [111,112].

Figure 6 shows two possible implementations of the plasmonic boom structures. The plasmonic boom instability [19,20] occurs when the electron velocity exceeds the plasma velocity. It is similar to the sonic boom occurring when a jet achieves a supersonic velocity. In a plasmonic crystal, such instability should be very effective if the electron drift velocity repeatedly goes higher and lower than the plasma velocity. In the structure shown in Figure 6a, the plasma frequency is modulated via having the periodic pattern of varying electron densities. In contrast, the structure shown in Figure 6b corresponds to the same plasma frequency spectrum in all the regions. However, the electron drift velocity is higher in narrower regions and smaller in the wider regions. Using narrow protruding regions called plasmonic stubs allows for the phase control in plasmonic 1D, 2D, and 3D plasmonic crystals, schematically shown in Figure 7. The stubs could be gated as shown in Figure 6 and, therefore, tunable, allowing for the optimized phase relations [113].

The tunable transmission by the grated gate structure was described in [114]. The transmission minima at the fundamental plasma frequency and its harmonics were observed in the temperature range from 4.2 K to 170 K. THz generation by a grating gate structure, due to a plasmonic boom instability was proposed, for the first time, in [19] and observed in [115].

**Figure 9:**
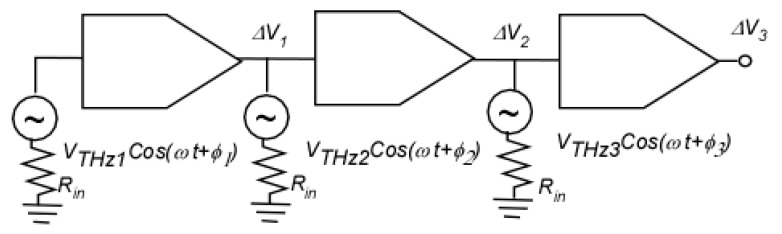
Schematic of the two-stage TeraFET amplifier detector with the THz signals applied to each stage with a phase shift.

**Figure 10:**
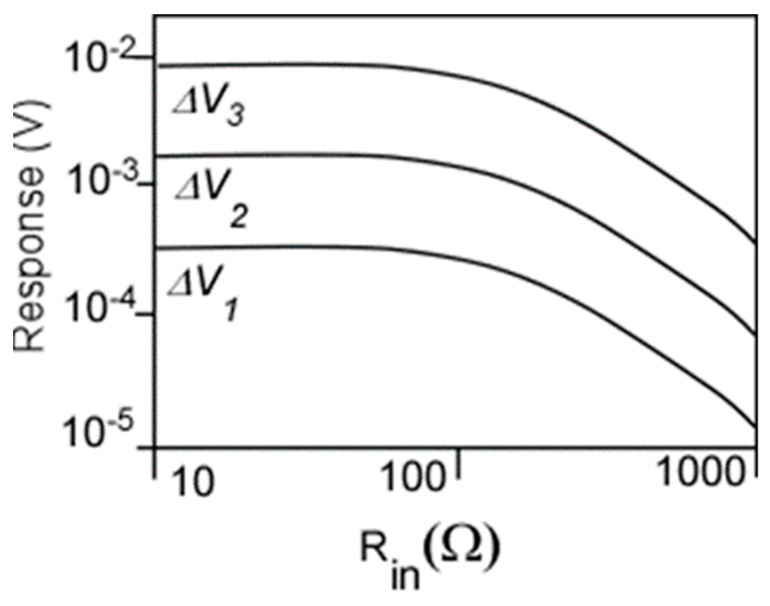
Drain response for each stage using harmonic balance (HB) and transient simulation as a function of the THz signal impedance with different schemes and approaches. ΔU_1_, ΔU_2_, and ΔU_3_ are the response at V_d1_, V_d2_, and V_d3_, respectively (see Figure 9).

The idea of adjusting the phase shifts in the unit cells of a plasmonic crystal for the vector detection of generation could be also applied to a travelling wave amplifier concept [45] (see Figure 9). Feeding the phase-shifted terahertz (THz) signal into the stages of a TeraFET amplifier dramatically increases the response (see Figure 10). The number of stages is only limited by the THz beam cross-section. As seen from Figure 10, this “traveling wave amplifier” approach enables orders of magnitude enhancement in the THz detector responsivity.

## 4. TeraFET Sources

As mentioned above, the state-of-the-art Si FET emitters using frequency multiplication generate in the order of a hundred of microwatts power in the 300 GHz range [106]. However, the TeraFET output power could be increased using a series connection of TeraFETs [107] and even more by using the plasmonic crystal TeraFETs discussed above with the estimated power output on the order ~100 mW [20]. Table 3 compares the performance of the existing THz electronic sources.

Terahertz plasmon amplifying graphene-channel transistors have been also proposed (see [128] and references therein.) Vacuum electronic sources could produce THz radiation in a large range of frequencies and output powers. Free electron lasers generated up to 500 kW peak power [129] in the range of 0.1 to 2.73 THz; BWO lamps generate from 50 mW to 10 kW power in the 0.2 to 0.65 THz range [130]; and klinotrons produce up to watts of power in the range 0.1 to 0.5 THz [131], with gyrotrons producing kilowatts of power in the THz range [132]. However, THz electronic sources, especially Si CMOS, have the highest potential for 6G communications in the 300 GHz band. The progress in developing this technology has been incremental but important. The Si CMOS and BiCMOS plasmonic sources, even in the 300 GHz band, where plasmons quickly decay, have a promise of orders of magnitude improvement by developing circuits with matched phases between the stages (see reference [45] for the description of this concept).

## 5. Other TeraFET Applications

**Figure 11:**
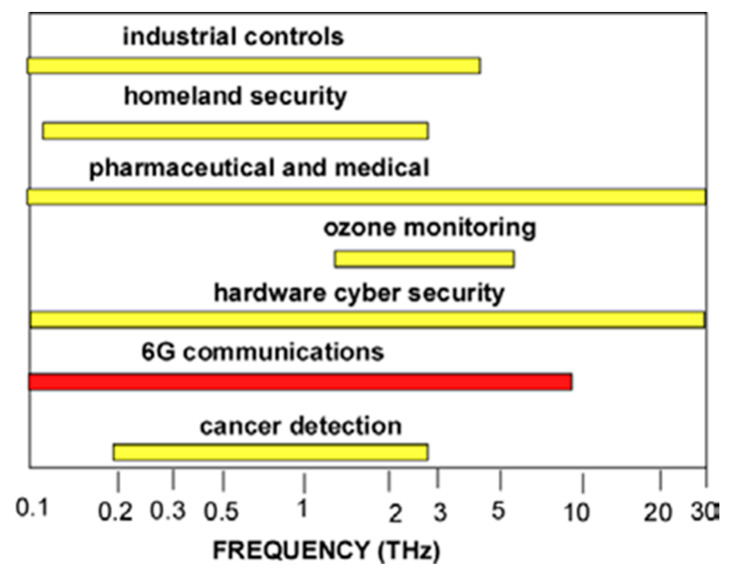
THz frequency ranges for different applications.

TeraFET technology will also enable THz applications including astronomical science [133,134,135], earth observation [136], sensing [137,138,139], chemical analysis [140,141], homeland security, concealed weapon and explosive detection [142,143], industrial controls [144,145], compact radars [146,147], structural integrity testing, spacecraft tiles control [144], Internet of Things (IoT), biotechnology [148,149,150], medicine [151,152], including cancer diagnostics [153], and non-destructive VLSI testing during the manufacturing process [154] and in-situ checking of the THz scans of chips [155,156,157,158,159]. Artificial intelligence processing of the VLSI THz scans allows distinguishing between genuine and fake VLSI for hardware cyber security applications [158]. Figure 11 shows the THz frequency ranges for different applications of the THz technology.

The THz range for 6G applications is extended to 10 THz to include communications in space [133] and between computer chips [160]. The development of 6G THz communication technology will be very beneficial for all other THz applications.

## 6. Conclusions

Short-channel TeraFETs and TeraFET plasmonic crystals have great potential for supporting transformational 6G communications technology in the 300 GHz band and beyond. The TeraFET physics involves ballistic or quasi-ballistic transport with the channel dimensions being smaller than or close to the electron mean free path in the TeraFET channel. GaAs plasmonic THz imaging arrays have already been commercialized [161]. Si CMOS deep submicron TeraFETs have also demonstrated excellent performance. This technology could support 300 GHz line-of-sight detectors, travelling wave amplifiers, spectrometers, and generators. III-N-based TeraFETs could find applications in the 6G communication towers because of their potential of delivering a higher power [162,163]. In a longer run, TeraFETs based on novel plasmonic materials, such as graphene, graphene-based heterojunctions, and p-diamond, might compete with GaAs and Si CMOS TeraFETs, and might even extend THz communications to higher THz frequencies.

## Figures and Tables

**Table 1:** TeraFET detector performance at sub-THz and THz frequencies (data from [82]).

Detector Type	Frequency (THz)	Noise Equivalent Power (pW/Hz^1/2^)
AlGaN/GaN TeraFETs	0.49–0.65	25–31
AlGaN/GaN TeraFETs	0.7–0.9	30–50
65 nm Si CMOS	0.8–1	100
65 nm Si CMOS	0.65	17
65 nm Si CMOS	0.72	14
90 nm Si CMOS	0.6	48–70
130 nm Si CMOS	0.26	8.4

**Table 2:** Performance of THz detection devices (data from [101,102,103]).

THz Detector	Mechanism	Speed	Operating Temperature (K)	Responsivity (kV/W or A/W)	NEP (pW/Hz^−1/2^)
Golay cell	Thermal	Slow	300	10–100 kV/W	~100
Bolometer	Thermal	Slow	4.2	~100 kV/W	~0.1
Schottky diode	Rectification	Fast	300	0.1 to 1.5 kV/W	2.7 to 40
GaN TeraFET	Plasmonic	Fast	300	1.1 kV/W	40
Grated Gate TeraFET	Plasmonic	Fast	300	2.2 to 23 kV/W	0.5 to 50
Resonant tunnelling diode	Resonant tunnelling	Fast	300	7.3 A/W	7.7
Resonant tunnelling diode	Resonant tunnelling	Fast	300	0.9 kV/W	2.5

**Table 3:** THz electronic sources operating at 300 K unless stated otherwise (see also [116,117,118,119,120,121,122,123,124,125,126,127]).

THz Emitter	Frequency (THz)	Output Power (mW) 300 K	DC Power (mW)	Efficiency (%)	Reference
40 nm Si CMOS	0.266	0.69	1790	0.039	[7]
GaAs pHEMT	0.144–0.432	0.063 @300 K 0.278 @77 K	180 (estimated)	0.1	[60]
130 nm SiGe HBTs	0.25	7.08	1960	0.36	[116]
130 nm SiGe BiCMOS	0.34	1.02	1700	0.87	[117]
Schottky diode frequency multipliers	0.05–520	1900–200	-	38–5	[123]
Gunn diodes	0.1–0.3	0.05–0.023	-	-	[124]
IMPATT diodes	0.1–300	400–10	-	-	[125]
Resonant tunnelling diode array	1–1.98	0.7	-	-	[126]
THz plasmon-emitting graphene-channel transistor	1–7.6	0.01 @5.2 THz 0.001 @7.6 THz @100 K	-	-	[127]

## Data Availability

Not applicable.
